# Does constipation affect the effectiveness of washed microbiota transplantation in treating autism spectrum disorders?

**DOI:** 10.3389/fnins.2025.1602681

**Published:** 2025-07-23

**Authors:** Zihao Pan, Zheng Gao, Junyi Chen, Yongxi Quan, Jiating Xu, Xiaofeng Liang, Wenrui Xie, Xingxiang He, Lihao Wu

**Affiliations:** ^1^Department of Gastroenterology, The First Affiliated Hospital of Guangdong Pharmaceutical University, Guangzhou, China; ^2^Research Center for Engineering Techniques of Microbiota-Targeted Therapies of Guangdong Province, Guangzhou, China

**Keywords:** washed microbiota transplantation, autism, clinical efficacy, constipation, sleep disorders, intestinal barrier function

## Abstract

**Purpose:**

Washed microbiota transplantation (WMT) has been shown to improve the symptoms of Autism Spectrum Disorder (ASD). It’s currently unclear whether the presence of constipation affects the efficacy of WMT in children with ASD. This study aims to investigate whether constipation affects the efficacy of WMT in children with ASD.

**Patients and methods:**

To investigate the efficacy of WMT for ASD, we conducted a retrospective analysis of changes in ASD-related symptoms, sleep disturbances, gastrointestinal manifestations, intestinal barrier integrity, and gut microbiota composition in 103 ASD patients undergoing WMT. They were divided into two groups according to whether constipation was present or not before treatment.

**Results:**

1. Aberrant Behavior Checklist (ABC), Childhood Autism Rating Scale (CARS), and Sleep Disturbance Scale for Children (SDSC) scores in the non-constipation and constipation groups decreased with an increase in the number of WMT treatments. 2. Comparison of two groups: ABC scores in the non-constipation group decreased more after the first WMT course, whereas ABC scores in the constipation group decreased more after two WMT courses. 3. Intestinal Barrier Function: D-lactate levels decreased more in the constipation group after the first two courses. In general, WMT treatment had no significant effect on intestinal barrier function in patients with ASD. 4. Effect of WMT on constipation: As the number of WMT courses increased, Bristol Stool Form Scale (BSFS) scores in constipation group gradually approached 4. 5. Constipation group had lower microbial diversity than non-constipation group at baseline. After one course of WMT, constipation group showed an obvious increase in microbial diversity and a significant increase in the relative abundance of Bifidobacteria compared to non-constipation group.

**Conclusion:**

Post WMT, core symptoms and sleep disorders were significantly improved in both groups. Feces returned to normal shape in the constipation group. A difference in efficacy between the two groups was observed in early stages, but after multiple courses of WMT no difference in efficacy was noted. Although in the short-term, children with ASD and comorbid constipation showed a significant increase in microbial diversity after receiving WMT, mid-term outcomes indicate that constipation does not affect the efficacy of WMT in treating ASD.

## 1 Introduction

Autism Spectrum Disorder (ASD) is a common neurodevelopmental disorder, with core symptoms including social and communication difficulties, repetitive behaviors and language dysfunction (Lord et al., 2020). Additionally, patients with ASD commonly have gastrointestinal disturbances, including symptoms such as constipation, diarrhea, and abdominal pain (Ibrahim et al., 2009). According to research statistics, the prevalence of gastrointestinal symptoms in patients with ASD ranges from 9 to 91%. Among these, constipation is the most common gastrointestinal comorbidity in patients with ASD, with an incidence rate of approximately 33.2% (Madra et al., 2020; Zhu et al., 2017). A recent study suggest that gastrointestinal issues are associated with ASD core symptoms as well as increased severity of certain comorbid symptoms, such as irritability and aggressive behavior (Hung and Margolis, 2024; Yang et al., 2023). Another cross-sectional study supports this perspective, finding that the prevalence of gastrointestinal symptoms in patients with ASD is that twice that of typically developing children. Moreover, patients with ASD with gastrointestinal symptoms exhibit more severe repetitive behaviors and emotional problems (Zhu et al., 2017). Therefore, gastrointestinal symptoms exhibit a statistically significant positive correlation with the severity of core ASD symptoms. Furthermore, recurrent gastrointestinal symptoms can lead to a decrease in gut microbiota diversity and damage to intestinal mucosal barrier, preventing certain interventions for ASD, including rehabilitation training, psychotherapy, and pharmacotherapy from achieving the desired therapeutic effect (Mitchell et al., 2023; Morrill et al., 2024). This bidirectional interplay between gastrointestinal symptoms and mucosal barrier dysfunction exacerbates ASD-related symptomatology, forming a vicious cycle that amplifies disease severity.

Multidirectional and complex interactions exist between ASD, microbiota and constipation. Compared to typically developing children, the ratio of Bacteroidetes to Firmicutes in the gut of children with ASD is generally lower (Lewandowska-Pietruszka et al., 2023). Children with lower abundance of Bacteroidetes are more likely to have both ASD and constipation. In children who have been diagnosed with ASD, low abundance of Firmicutes in the gut may be one of the primary causes of constipation (Fu et al., 2021). This suggests the existence of a pathological network involving core ASD symptoms, altered gut microbiota, and gastrointestinal dysfunction.

Meanwhile, increased intestinal permeability is considered a key link in the “gut-brain axis” in the etiology of autism (Dargenio et al., 2023). Increased intestinal permeability may lead to toxic substances entering the bloodstream, causing a systemic inflammatory response, affecting the diversity of the gut microbiota, and disrupting the balance between beneficial and harmful microbiota, which in turn aggravates the core symptoms of ASD and gastrointestinal symptoms (Al-Ayadhi et al., 2021; Wang et al., 2020). Studies have found that compared to normal subjects, patients with ASD exhibit a higher prevalence of abnormal intestinal permeability. Additionally, their fecal calprotectin levels are elevated (Berni Canani et al., 2004; de Magistris et al., 2010). These findings suggest that maintaining a healthy and balanced gut microbiota and the integrity of the intestinal barrier is crucial for patients with ASD.

Fecal Microbiota Transplantation (FMT) is a microbial therapeutic technique that involves transplanting the microbiota from fresh stool of healthy donors into the intestines of patients. This therapeutic technique aims to regulate patients’ gut microbiota environment, restore a normal gut microbiota, and improve intestinal barrier function (Ye et al., 2024; Zhang et al., 2018). Washed Microbiota Transplantation (WMT), an advanced form of FMT, involves additional processing of stool samples using an automated microbiota separation system. This process removes most fecal particles, parasitic eggs, fungi and other harmful substances, enhancing safety while maintaining therapeutic efficacy (Zhang et al., 2020).

Previous research from our team has demonstrated that FMT can improve ASD patient’s core symptoms, sleep quality and accompanying gastrointestinal symptoms by modulating the gut microbiota (Liu et al., 2023; Pan et al., 2022). However, the relationship between autism, the gut microbiota and gastrointestinal disorders remains unclear. Constipation, as the most common comorbidity, has yet to be thoroughly investigated regarding its potential impact on the treatment of children with ASD. This research aims to evaluate the impact of constipation symptoms on the clinical efficacy of WMT in the treatment of ASD, as well as its effects on intestinal barrier function and gut microbiota. It seeks to determine whether patients with ASD with constipation achieve the same therapeutic outcomes from WMT as those without constipation. The goal is to provide a scientific and effective WMT treatment plan for patients with ASD who also present with constipation.

## 2 Subjects and methods

This is a retrospective study conducted at the First Affiliated Hospital of Guangdong Pharmaceutical University. This study has been approved by the Ethics Committee of the First Affiliated Hospital of Guangdong Pharmaceutical University [Approval No. (2024) IIT; Turroni et al., 2021]. The study protocol adheres to the principles of the Declaration of Helsinki. Informed consent was obtained from the guardian of each child. Clinical Trial Registration was also obtained for the study, Chinese Clinical Trial Registry, ChiCTR2400094256. Registered 19 December 2024, www.chictr.org.cn.

We continuously collected data from 103 children with ASD who received at least two courses of WMT treatment at the First Affiliated Hospital of Guangdong Pharmaceutical University between January 2020 and February 2024.

Inclusion criteria: (I) Age between 2 and 18 years; (II) No restrictions on gender; (III) ASD diagnosed children per DSM-5 criteria; (IV) No prior history of undergoing WMT.

Exclusion criteria: (I) Use of antibiotics or probiotics within one month prior to receiving WMT treatment; (II) Diagnosed with gastrointestinal diseases requiring urgent treatment, such as inflammatory bowel disease, gastrointestinal neoplasms or celiac disease; (III) Diagnosed with severe heart, brain, lung, liver or kidney diseases.

At baseline, 103 children with ASD were divided into the constipation group (C-ASD) and the non-constipation group (NC-ASD) according to the Rome IV diagnostic criteria. There were 59 participants in the non-constipation group and 44 participants in the constipation group. In the non-constipation group, 59 participants completed two courses of WMT, 49 completed three courses, 36 completed four courses, 27 completed five courses, and 12 completed six courses. In the constipation group, 44 participants completed two courses of WMT, 37 completed three courses, 25 completed four courses, 17 completed five courses, and 9 completed six courses. We also collected fecal specimens from 59 participants (38 in the non-constipation group and 21 in the constipation group) before treatment and after the first course of WMT for 16sRNA sequencing.

### 2.1 WMT treatment procedure

In this study, WMT treatment was conducted based on the recommendations provided in the Nanjing Consensus on Methodology of Washed Microbiota Transplantation.

Healthy donors for WMT underwent comprehensive screening conducted by licensed clinicians. The donor screening process included questionnaires, interview screenings, physical examinations, laboratory tests, and monitoring screenings. Candidates who passed the interview screening were required to undergo laboratory tests on blood, urine, and stool samples to exclude infectious diseases and potential microbiota dysbiosis-related disorders. Informed consent was obtained from the parents or guardians of donors prior to stool donation.

A homogeneous fecal suspension was prepared with a ratio of 100 g of feces to 500 mL of normal saline. The fecal suspension was then filtered using a microfiber filtration instrument (GenFMTer; FMM Medical, Nanjing, China) to remove food particles, inflammatory substances, and fungi, enhancing the safety of the microbiota suspensions. After standardized preparation of the bacterial suspension, the culture was transported to the ward via a dedicated transport container and delivered by trained personnel. The bacterial suspension was then rewarmed to 37°C using a constant temperature water bath and administered to patients through the Transendoscopic enteral tubing (TET) within 2 h. This timeframe was critical to minimize excessive exposure of anaerobic bacteria and prevent dynamic growth in a carrier solution environment distinct from intestinal fluid, thereby preserving the structural integrity of viable microbial communities.

For sample preservation, 2 mL of supernatant samples (post-third wash and centrifugation) and washed fecal microbiota samples were aliquoted into sterile EP tubes and stored at −80°C in a low-temperature freezer for safety traceability. All fecal samples were meticulously labeled with detailed identifiers.

All patients were instructed to consume a liquid diet the day before WMT treatment to facilitate bowel preparation. Children with constipation were advised to take a moderate amount of lactulose the day before treatment to soften stools. Prior to colonoscopy, an anesthesiologist will evaluate the child’s baseline condition, and the parents will sign the informed consent form. Subsequently, under general anesthesia, the endoscopist will advance the TET tube terminal to the cecum under colonoscopic guidance. The proximal end will be secured to the intestinal wall using disposable titanium clips, while the distal end will be fixed to the right hip joint. Each WMT treatment involved the administration of a fecal suspension containing approximately 5.0 × 10^13^ viable bacteria, delivered daily (60–90 mL) for 6 consecutive days via the TET tube. In this study, fresh fecal microbiota suspension from healthy donors was used for WMT treatment. The suspension was transplanted into patients who were required to remain in the right lateral position for more than 2 h after the procedure.

Additionally, all patients were advised to avoid consuming spicy and irritating foods for at least 2 days before accepting WMT treatment. Transplantation was administered once daily for 6 consecutive days, completing one full course of WMT. The interval between each WMT course was set at 1 month.

All pediatric patients received continuous safety oversight from a multidisciplinary medical team comprising pediatricians and gastroenterologists throughout the WMT treatment period. Nurses conducted vital sign assessments every 12 h and performed routine symptom evaluations. Comprehensive pre-treatment laboratory evaluations, including complete blood count, liver/kidney function tests, and electrolyte panel analysis, were mandatory prior to WMT initiation. In the event of severe adverse reactions such as hyperthermia exceeding 38.0°C or profuse diarrhea-induced hypokalemia, WMT administration would be temporarily suspended following specialist evaluation.

### 2.2 Data collection and definitions

Patients’ demographic data (gender, BMI, age), ASD symptoms, sleep status, gastrointestinal symptoms, and intestinal barrier serological markers were collected from the electronic system and paper medical records. Autism Behavior Checklist (ABC) and Childhood Autism Rating Scale (CARS) were used to assess ASD symptoms. The ABC scale evaluates autism in five common domains: sensory, communication, body movement, language, and self-care. A score higher than 53 suggest a high likelihood of ASD (Li et al., 2021a; Li et al., 2021b). The CARS scale involves 15 items, it is mainly used to diagnose the severity of ASD symptoms (30–36, mild to moderate; > 36, severe) (Schopler et al., 1980). The Sleep Disturbance Scale for Children (SDSC) was used to assess the sleep quality of the children. A score higher than 39 indicates significant sleep disturbance, with higher scores reflecting poorer sleep quality (Chen et al., 2022). The CARS scale was completed by a pediatrician who was blinded to the treatment, while the ABC and SDSC scales were filled out by the parents of the children. Intestinal barrier function was assessed by measuring serum levels of Diamine Oxidase (DAO), D-lactate, and Lipopolysaccharide (LPS). When serum levels of DAO > 10 U/L, D-lactate > 15 mg/L, and LPS > 20 U/L, it indicates intestinal mucosal damage and increased intestinal permeability (Ye et al., 2024). Constipation was diagnosed based on the Rome increased intestinal (Mulhem et al., 2022). Stool consistency was assessed using the Bristol Stool Form Scale (BSFS), which classifies stool into 7 types, each reflecting different digestive and excretory states (1 = very hard, 7 = liquid, 3–5 = normal stool form) (Lemay et al., 2021). Relevant assessments and evaluations were conducted at the baseline and approximately 1 month after each treatment course.

### 2.3 Fecal sample collection and intestinal microbiota testing procedure

Among the 103 participants, fresh fecal samples were collected from 59 ASD subjects. The stool samples were gathered both before the first course of WMT treatment and 1 month after the treatment. They were immediately frozen at -80They wetorage.

First, total DNA was extracted from the fecal samples, and the 16 S V3-V4 variable region was amplified by PCR to construct the sequencing library. High-throughput sequencing was performed using the Illumina MiSeq PE300 platform (Majorbio Bio-Pharm Technology Co., Ltd., Shanghai)). The raw sequencing data were then uploaded to the NCBI database for further analysis. The raw sequencing reads were quality-controlled using Trimmonatic software and assembled using FLASH software Operational taxonomic units (OTUs) were clustered with UPARSE software based on 97% sequence similarity. Taxonomic annotation for each sequence was performed using the RDP classifier, with comparison against the Silva database, and a confidence threshold of 70% was applied for the classification.

### 2.4 Statistical analysis

Statistical analysis was performed using SPSS 27.0 and Prism 9 software. Continuous variables were presented as mean ± standard deviation if they followed a normal distribution, while median and interquartile ranges were used for non-normally distributed continuous variables. Categorical variables were expressed as frequencies or percentages. Fisher’s exact test or the chi-square test was used to analyze independent categorical variables. Significant differences between two independent groups were assessed using independent samples *t*-test (normal distribution) or the Mann-Whitney U test (non-normal distribution). Paired data were analyzed using the paired samples *t*-test (normal distribution) or Wilcoxon signed rank test (non-normal distribution). Correlations between factors were analyzed using Spearman correlation analysis. Differences were considered significant when the *P*-value was < 0.05.

## 3 Results

### 3.1 Clinical characteristics of children undergoing WMT

Demographic and clinical characteristics of the study subjects are presented in Table 1. There were no statistically significant differences in baseline data between the two groups of ASD children before treatment (Table 1).

**TABLE 1:** Baseline data were compared between the two groups of patients.

	Non-constipation group (*n* = 59)	Constipation group (*n* = 44)	*P*
Gender (male)	55 (93.2%)	36 (81.8%)	0.07
Age	6.00 (5.00–8.00)	5.50 (4.00–7.00)	0.18
BMI (kg/m^2^)	15.7 (14.4–18.1)	15.7 (14.3–17.4)	0.73
ABC	68.00 (60.00–84.00)	67.5.00 (42.00–82.50)	0.278
CARS	35.21 ± 3.06	36.01 ± 4.29	0.346
SDSC	46.00 (39.25–54.50)	48.00 (36.50–64.50)	0.721
DAO (U/L)	19.57 (14.92–26.83)	20.01 (15.20–34.79)	0.357
D-lactate (U/L)	31.33 (20.50–38.40)	33.81 (25.12–39.38)	0.193
LPS (U/L)	20.57 ± 6.05	22.75 ± 9.87	0.351

Data are expressed as mean ± standard deviation, median (interquartile range), or n (%). BMI, Body mass index; ABC, Aberrant Behavior Checklist; CARS, Childhood Autism Rating Scale; SDSC, Sleep Disturbance Scale for Children; DAO, Diamine Oxidase; LPS, Lipopolysaccharide.

Among the 103 children with ASD, only 6 (5.8%) experienced adverse symptoms during the first treatment course of WMT, including fever (*n* = 2), diarrhea (*n* = 3), and abdominal pain (*n* = 1). All adverse reactions were mild in severity and resolved with symptomatic treatment, with no severe adverse events reported.

### 3.2 Effects of WMT on core symptoms and sleep quality in ASD

**FIGURE 1:**
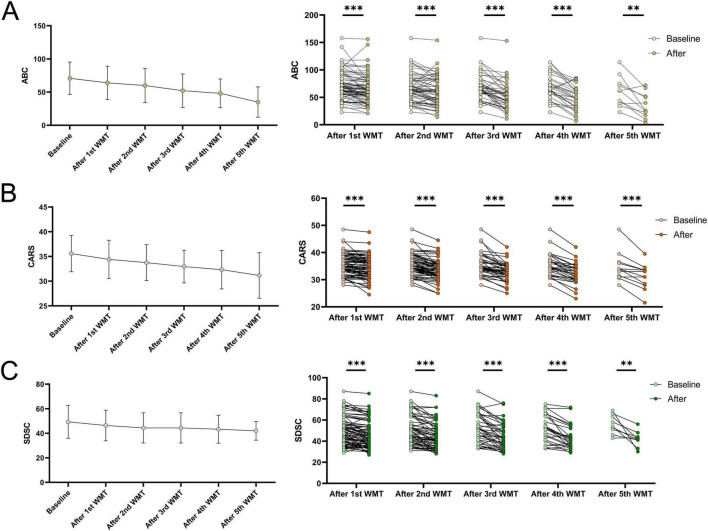
The effect of WMT on core symptoms of ASD and sleep quality are represented by changes in ABC **(A)**, CARS **(B)**, and SDSC **(C)** scores before and after WMT treatment. The line graph illustrates the mean and standard deviation of the data. Paired data after each course of WMT treatment were compared to their baseline levels, with statistical analysis performed using paired sample *t*-test of Wilcoxon signed-rank tests. *P < 0.05; **P < 0.01; ***P < 0.001; ns *P* > 0.05.

The ABC scale, CARS scale, and SDSC scale were used to assess the effects of WMT on core symptoms and sleep quality in 103 ASD children (Figure 1). The mean ABC scores at baseline and after each WMT course were 70.84 ± 24.33, 64.06 ± 24.95, 59.94 ± 25.77, 52.20 ± 24.93, 48.16 ± 21.32, 35.00 ± 21.78. The mean CARS scores at baseline and after each WMT course were 35.59 ± 3.67, 34.42 ± 3.85, 33.75 ± 3.62, 32.97 ± 3.26, 32.34 ± 3.83, 31.17 ± 4.40. The mean SDSC scores at baseline and after each WMT course were 49.34 ± 13.28, 46.34 ± 12.42, 44.34 ± 12.21, 44.38 ± 12.17, 43.30 ± 11.17, 42.00 ± 7.18. Comparing each WMT course score with baseline levels showed that ABC, CARS, and SDSC scores decreased with the increase in WMT courses. The scores on all three scales were significantly lower than the baseline levels after WMT treatment (*P* < 0.05), indicating improvement in ASD core symptoms and sleep quality as WMT progressed.

### 3.3 Impact of WMT on constipation symptoms in children with ASD from the constipation group

**FIGURE 2:**
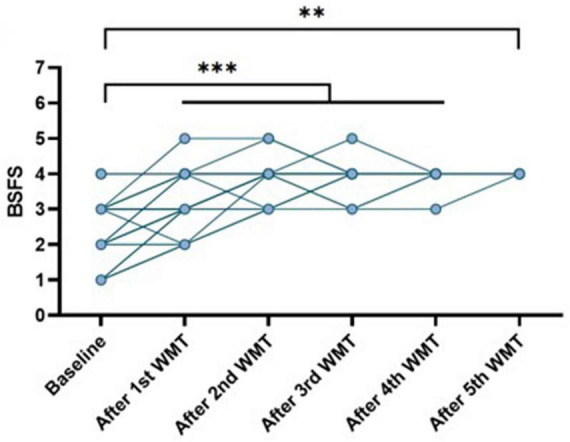
The impact of WMT on constipation symptoms. *P < 0.05; **P < 0.01; ***P < 0.001; ns *P* > 0.05.

The BSFS was collected for 44 children with ASD from the constipation group, both at baseline and after each course of WMT treatment. Our study found that at baseline, the BSFS scores for children with ASD in the constipation group ranged from 1 to 4. With the progression of WMT treatment, the BSFS scores gradually approached 4, indicating a return to normal stool form (*P* < 0.05) (Figure 2).

### 3.4 Impact of WMT on intestinal barrier function in children with ASD

**FIGURE 3:**
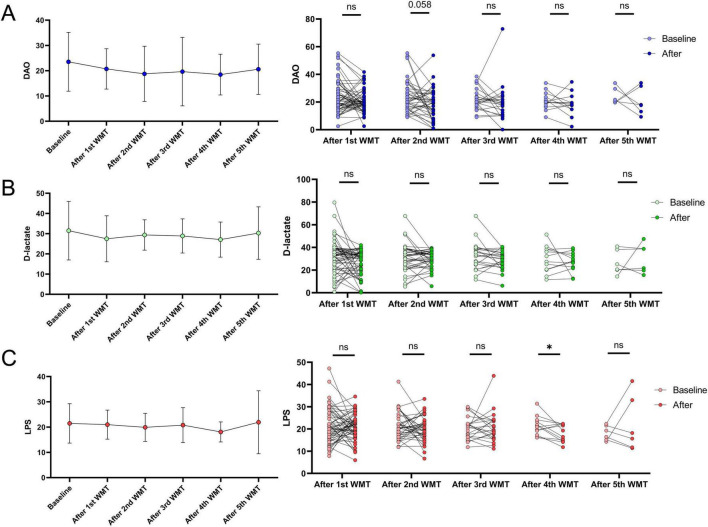
The effects of WMT on intestinal barrier function in ASD are represented by changes in blood DAO **(A)**, D-lactate **(B)**, and LPS **(C)** levels before and after WMT treatment. The line graph displays the mean and standard deviation of the data. Paired data after each WMT course were compared to their respective baseline levels using paired sample *t*-tests or Wilcoxon signed-rank tests for statistical analysis.

Investigating the effect of WMT on intestinal barrier function in 103 children with ASD, the mean blood DAO levels at baseline and after each WMT course were 23.54 ± 11.58, 20.73 ± 7.93, 18.77 ± 10.81, 19.63 ± 13.26, 18.45 ± 7.77, 20.59 ± 9.08 (Figure 3A). The mean blood D-lactate levels at baseline and after each WMT course were 31.47 ± 14.36, 27.50 ± 11.31, 29.38 ± 7.43, 28.87 ± 8.28, 27.06 ± 8.36, 30.31 ± 11.83 (Figure 3B). The mean blood LPS levels at baseline and after each WMT course were 21.45 ± 7.74, 20.98 ± 5.72, 19.89 ± 5.51, 20.77 ± 6.76, 18.09 ± 3.79, 21.90 ± 11.37 (Figure 3C).

Each intestinal barrier-related indicator after each WMT course was compared to its baseline level. The results showed that although endotoxin levels were lower than baseline after the fourth WMT course (*P* < 0.05), there were no significant differences after the first, second, third, and fifth WMT courses (Figure 3C). Similarly, no significant differences were observed in blood DAO and D-lactate levels after WMT treatment compared to baseline across all five courses (Figures 3A,B). Overall, WMT treatment had no significant impact on the intestinal barrier function of children with ASD.

### 3.5 Comparison of core symptoms and sleep quality between non-constipation group and constipation groups following WMT treatment

Scores on the ABC, CARS, and SDSC decreased with increasing numbers of WMT treatments in both the non-constipation and constipation groups (Table 2).

**TABLE 2:** Paired data from each WMT course for both groups are compared with their respective baseline levels in table.

ABC	1st WMT	2nd WMT	3rd WMT	4th WMT	5th WMT
Non-constipation group	60.00 (50.00, 70.00) vs. 68.00 (60.00, 84.00)	59.34 ± 23.49 vs. 61.94 ± 17.97	51.50 ± 20.71 vs. 69.00 ± 16.10	50.00 (33.00, 62.00) vs. 64.00 (61.00, 75.00)	43.20 ± 22.20 vs. 63.00 ± 11.94
P	*P* < 0.05	*P* > 0.05	*P* < 0.05	*P* < 0.05	*P* > 0.05
Constipation group	65.00 (43.75, 82.25) vs. 67.50 (42.00, 82.50)	53.00 (37.50, 83.50) vs. 68.00 (43.00, 90.00)	48.00 (32.00, 65.75) vs. 67.00 (42.50, 83.50)	44.42 ± 22.50 vs. 66.33 ± 31.18	28.17 ± 22.94 vs. 57.50 ± 36.57
P	*P <* 0.05	*P <* 0.05	*P <* 0.05	*P <* 0.05	*P <* 0.05
**CARS**	**1st WMT**	**2nd WMT**	**3rd WMT**	**4th WMT**	**5th WMT**
Non-constipation group	33.98 ± 3.83 vs. 35.21 ± 3.06	33.57 ± 3.08 vs. 35.06 ± 3.06	33.25 (30.25, 35.00) vs. 35.00 (33.00, 36.50)	31.68 ± 3.24 vs. 34.76 ± 3.33	30.92 ± 3.02 vs. 33.58 ± 2.40
P	*P* < 0.05	*P* < 0.05	*P* < 0.05	*P* < 0.05	*P* < 0.05
Constipation group	34.91 ± 3.91 vs. 36.01 ± 4.29	33.98 ± 4.29 vs. 35.97 ± 4.66	33.48 ± 3.74 vs. 36.20 ± 4.76	33.38 ± 4.72 vs. 35.83 ± 5.36	31.42 ± 6.10 vs. 35.83 ± 7.29
P	*P* < 0.05	*P* < 0.05	*P* < 0.05	*P* < 0.05	*P* < 0.05
**SDSC**	**1st WMT**	**2nd WMT**	**3rd WMT**	**4th WMT**	**5th WMT**
Non-constipation group	43.73 ± 10.07 vs. 48.10 ± 11.01	41.00 (33.00, 48.25) vs. 45.50 (39.00, 52.25)	41.09 ± 10.10 vs. 48.73 ± 11.25	40.06 ± 8.44 vs. 48.53 ± 11.85	45.80 ± 6.38 vs. 53.20 ± 9.26
P	*P* < 0.05	*P* > 0.05	*P* < 0.05	*P* < 0.05	*P* > 0.05
Constipation group	46.00 (36.00, 62.50) vs. 48.00 (36.50, 64.50)	43.00 (36.00, 60.00) vs. 53.00 (41.00, 66.00)	48.39 ± 13.83 vs. 57.11 ± 15.68	48.80 ± 13.94 vs. 58.40 ± 12.62	37.25 ± 6.80 vs. 59.25 ± 8.26
P	*P* > 0.05	*P* < 0.05	*P* < 0.05	*P* < 0.05	*P* < 0.05

Values in table are expressed as (pre-treatment data vs. post-treatment data). Statistical analysis was performed using paired sample *t*-tests or Wilcoxon signed-rank tests, with *P* < 0.05 considered statistically significant. WMT, Washed Microbiota Transplantation; ABC, Aberrant Behavior Checklist; CARS, Childhood Autism Rating Scale; SDSC, Sleep Disturbance Scale for Children.

**FIGURE 4:**
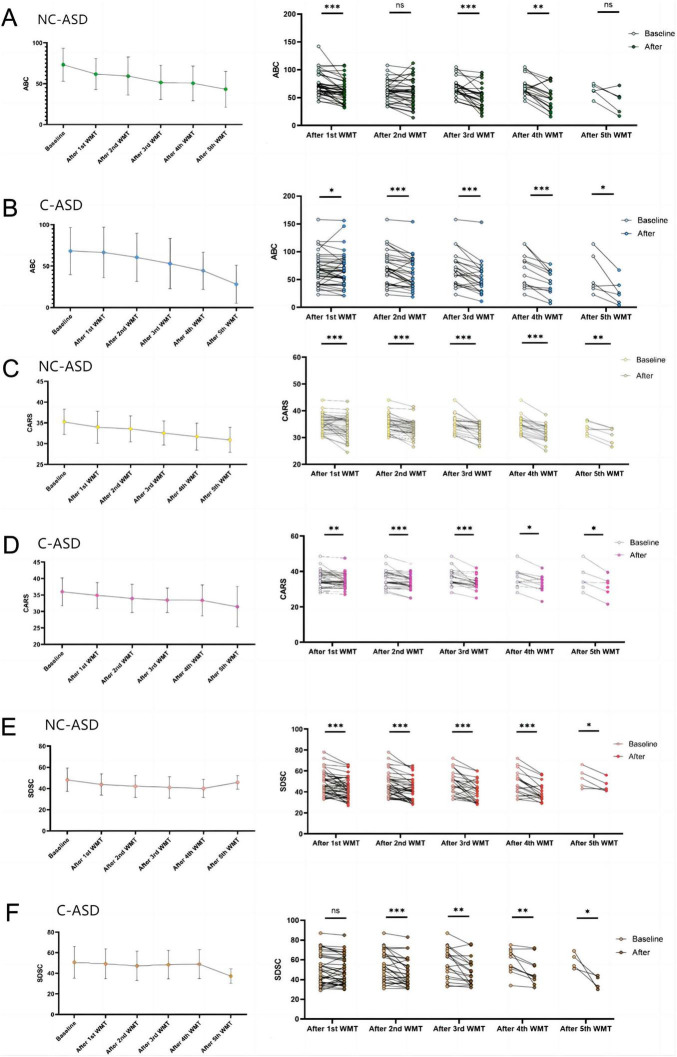
The effects of WMT treatment on core symptoms and sleep quality in non-constipation and constipation groups are represented by changes in ABC, CARS, and SDSC scores before and after treatment. The line graphs display the mean and standard deviation of the data. Paired data from each WMT course both groups were compared with their respective baseline levels using paired sample *t*-tests or Wilcoxon signed-rank tests for statistical analysis. *P < 0.05; **P < 0.01; ***P < 0.001; ns *P* > 0.05. **(A)** ABC scores in non-constipation group, **(B)** ABC scores levels in constipation group, **(C)** CARS scores in non-constipation group, **(D)** CARS scores in constipation group, **(E)** SDSC scores in non-constipation group, **(F)** SDSC scores in constipation group.

ABC scores: After the first, third, fourth WMT course, the ABC scores of non-constipation group were significantly lower than baseline level (*P* < 0.05), with no significant differences after the second and fifth courses (Figure 4A). In the constipation group, ABC scores were significantly lower than baseline after all five WMT courses (*P* < 0.05) (Figure 4B).

CARS scores: In both non-constipation (Figure 4C) and constipation groups (Figure 4D), CARS scores showed a significant decrease compared to baseline after all five WMT courses (*P* < 0.05).

SDSC scores: In the constipation group, SDSC scores significantly decreased compared to baseline after the second, third, fourth, and fifth WMT courses (*P* < 0.05), while no significant difference was observed after the first course (Figure 4F). In the non-constipation group, SDSC scores significantly decreased compared to baseline after all five WMT courses (*P* < 0.05) (Figure 4E).

Comparing the difference in scores between each WMT course and baseline in both groups, the results showed a significant difference in ABC score changes after the first and second WMT courses. After the first course, the non-constipation group showed a greater score reduction [-8.00 (-14.50, 43.00) vs. s2.50 (-9.00,0.00), *P* < 0.05]. After the second course, the constipation group had a greater score reduction [-5.00 (-10.00, 2.00) vs. -11.00 (-22.00, -4.00), *P* < 0.05]. No significant differences were observed between the two groups after the third, fourth, and fifth courses (Table 3).

**TABLE 3:** The difference value (D) represents the score for each scale after each WMT course minus the baseline score.

The difference of ABC(D)	Non-constipation group	Constipation group	*P*
The 1st WMT (41:38)	−8.00 (−14.50, 43.00)	−2.50 (−9.00, 0.00)	0.004
The 2nd WMT (35:29)	−5.00 (−10.00, 2.00)	−11.00 (−22.00, 24.00)	0.010
The 3rd WMT (24:20)	−17.50 ± 18.01	−16.90 ± 15.78	0.908
The 4th WMT (19:12)	−20.00 (−28.00, 15.00)	−17.50 (−30.00, 14.00)	0.704
The 5th WMT (5:6)	−19.80 ± 18.02	−29.33 ± 24.95	0.495
**The difference of CARS(D)**	**Non-constipation group**	**Constipation group**	***P***
The 1st WMT (42:38)	−5.00 (−1.25, 0.00)	−7.50 (−1.13, 0.00)	0.860
The 2nd WMT (36:29)	−1.00 (−2.00, −0.50)	−1.50 (−3.38, −0.13)	0.485
The 3rd WMT (24:20)	−2.00 (−3.38, −1.00)	−2.00 (−4.25, −0.63)	0.943
The 4th WMT (19:12)	−3.50 ± 1.53	−2.48 ± 2.72	0.190
The 5th WMT (6:6)	−2.67 ± 1.40	−4.42 ± 3.51	0.297
**The difference of SDSC(D)**	**Non-constipation group**	**Constipation group**	***P***
The 1st WMT (40:37)	−4.08 ± 7.32	−2.00 ± 5.98	0.175
The 2nd WMT (34:27)	−5.50 (−9.75, −1.00)	−6.00 (−9.50, −1.00)	0.913
The 3rd WMT (22:18)	−6.89 ± 9.33	−9.14 ± 9.34	0.453
The 4th WMT (17:10)	−6.50 (−12.50, 2.00)	−10.00 (−11.50, −6.50)	0.386
The 5th WMT (5:4)	−9.67 ± 7.06	−22.33 ± 16.04	0.130

Statistical analysis was performed using independent sample *t*-tests or Mann-Whitney U tests, with *P* < 0.05 considered statistically significant. WMT, Washed Microbiota Transplantation; ABC, Aberrant Behavior Checklist; CARS, Childhood Autism Rating Scale; SDSC, Sleep Disturbance Scale for Children.

### 3.6 Comparison of intestinal barrier function between non-constipation group and constipation group after WMT treatment

**FIGURE 5:**
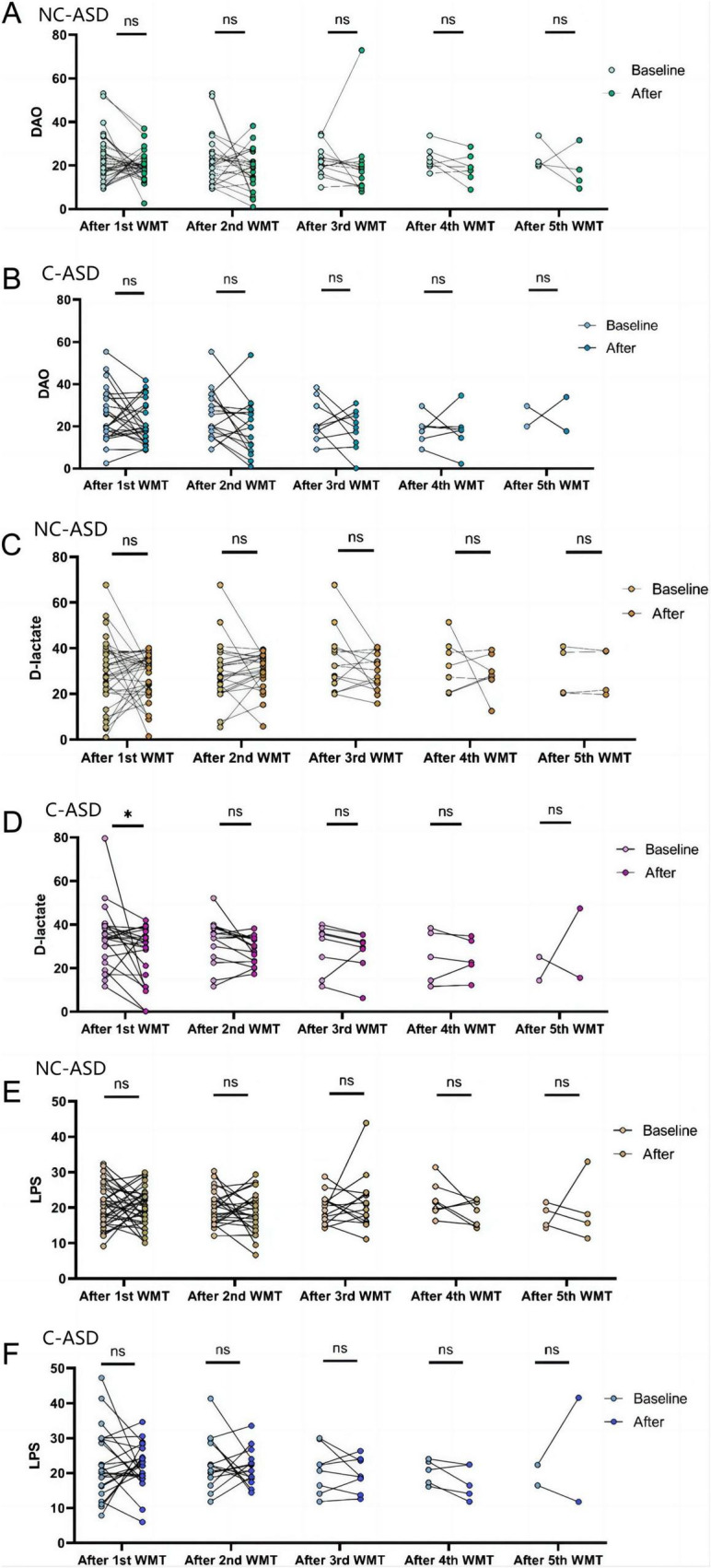
The effect of WMT treatment on intestinal barrier function in non-constipation and constipation groups is represented by changes in blood DAO, D-lactate, and LPS levels before and after treatment. Paired data for each WMT course were compared with baseline levels in both groups using paired *t*-tests or Wilcoxon signed-rank tests for statistical analysis. *P < 0.05; ns *P* > 0.05. **(A)** DAO levels in non-constipation group, **(B)** DAO levels in constipation group, **(C)** D-lactate levels in non-constipation group, **(D)** D-lactate level in constipation group, **(E)** LPS levels in non-constipation group, **(F)** LPS levels in constipation group.

Both constipation and non-constipation groups exhibited impaired intestinal barrier function at baseline (Figure 5). In constipation group, blood D-lactate levels were significantly lower than baseline after the first WMT course (*P* < 0.05) (Figure 5D). When comparing the difference between post-treatment and baseline blood D-lactate levels across one to five WMT courses, it was found that the constipation group experienced a greater reduction in blood D-lactate than non-constipation group during the first two courses [first course: e5.34 (-11.46, 1.92) vs. 3.00 (-7.73, 6.78); second course: e2.61 (-9.01, 1.50) vs. 2.81 (-3.94, 6.65), *P* < 0.05]. No significant differences were observed between the groups after the third, fourth, and fifth WMT courses. The blood DAO and LPS levels on both groups remained largely similar before and after the first to fifth WMT courses (Table 4). Due to the smaller sample size in the fifth WMT course, no statistical analysis was conducted for that session.

**TABLE 4:** The difference value (D) in the table refers to the post-treatment levels of blood DAO, D-lactate, and LPS minus baseline levels for each course of WMT.

The difference of DAO(D)	Non-constipation group	Constipation group	*P*
The 1st WMT (34:23)	−2.42 ± 11.86	−3.40 ± 13.60	0.775
The 2nd WMT (24:16)	−3.66 (−8.74, 5.62)	−5.61 (−14.14, 4.34)	0.440
The 3rd WMT (14:9)	−3.03 (−5.74, 1.31)	1.11 (−17.97, 7.18)	0.875
The 4th WMT (7:6)	−4.18 ± 5.91	−0.49 ± 10.78	0.450
The 5th WMT (4:2)	/	/	/
**The difference of D-lactate(D)**	**Non-constipation group**	**Constipation group**	***P***
The 1st WMT (34:23)	3.00 (−7.73, 6.78)	−5.34 (−11.46, 1.92)	0.044
The 2nd WMT (24:16)	2.81 (−3.94, 6.65)	−2.61 (−9.01, 1.50)	0.038
The 3rd WMT (14:9)	−2.59 (−9.17, 2.70)	−4.07 (−4.47, −2.80)	0.946
The 4th WMT (7:6)	1.20 (−10.90, 7.15)	−1.40 (−4.18, 3.93)	0.685
The 5th WMT (4:2)	/	/	/
**The difference of LPS(D)**	**Non-constipation group**	**Constipation group**	***P***
The 1st WMT (34:23)	−0.37 ± 8.12	−0.60 ± 10.84	0.927
The 2nd WMT (24:16)	−1.30 ± 7.18	−1.54 ± 9.11	0.926
The 3rd WMT (14:9)	−0.06 (−4.79, 6.13)	−0.53 (−5.51, 3.38)	0.539
The 4th WMT (7:6)	−3.70 ± 5.44	−2.91 ± 3.28	0.779
The 5th WMT (4:2)	/	/	/

Statistical analysis was performed using independent sample *t*-tests or Mann-Whitney U tests, with *P* < 0.05 considered statically significant. WMT, Washed Microbiota Transplantation; DAO, Diamine Oxidase; LPS, Lipopolysaccharide.

### 3.7 Analysis of gut microbiota diversity before and after WMT treatment in both non-constipation and constipation groups

The gut microbiota in fecal samples from the non-constipation group before and after WMT treatment were labeled as NC_ASD1 and NC_ASD2. The gut microbiota in fecal samples from the constipation group were labeled as C_ASD1 and C_ASD2. The Rank-Abundance curve can be used to explain two aspects of diversity: species richness and community evenness. Horizontally, species richness is reflected by the width of the curve-the wider the curve extends along the horizontal axis, the higher the species richness. The flatness of the curve reflects the community’s evenness-the flatter the curve, the more evenly the species are distributed across the community. The Shannon index is used to reflect the diversity of the gut microbiota, with higher values indicating greater community diversity.

**FIGURE 6:**
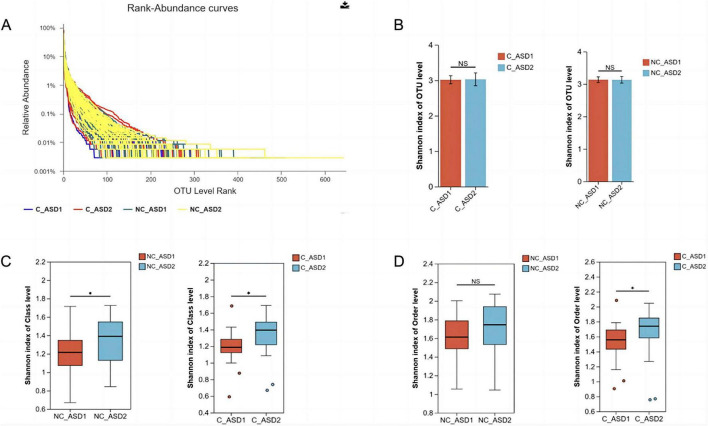
Microbial diversity changes in the two groups before and after WMT treatment. **(A)** Rank-Abundance curves: including pre-treatment and post-treatment for the non-constipation and constipation groups. **(B)** Alpha diversity analysis: the non-constipation and constipation groups. **(C)** Analysis of Shannon index at the level for both groups. **(D)** Analysis of Shannon index at the order level for both groups. *P < 0.05; ns *P* > 0.05.

The results from the Rank-Abundance curve indicate that the gut microbiota in NC_ASD2 patients has the highest species richness and evenness, followed by NC_ASD1, with C_ASD2 ranking third.C_ASD1 shows the lowest species richness and evenness (Figure 6A).

Alpha diversity analysis of the constipation and non-constipation groups showed that, at baseline, the gut microbiota in the constipation group displayed lower diversity than non-constipation group (Figure 6B). After one course of WMT treatment, the microbiota diversity in the constipation group increased, while it slightly decreased in the non-constipation group. However, neither change was statistically significant (*P* > 0.05) (Figure 6B).

At the class level, analysis showed that after one course of WMT treatment, both of the constipation and non-constipation groups experienced a significant increase in the Shannon index (*P* < 0.05) (Figure 6C). At the order level, only the constipation group demonstrated a significant increase in the Shannon index after one treatment course (*P* < 0.05) (Figure 6D). No significant differences were observed before and after treatment at the phylum, family, and genus levels for either group.

### 3.8 Comparison of the species composition and difference analysis of gut microbiota before and after WMT treatment between the non-constipation and constipation groups

Significance testing of inter-group species differences was conducted for each taxonomic level from phylum to genus before and after treatment in both groups.

**FIGURE 7:**
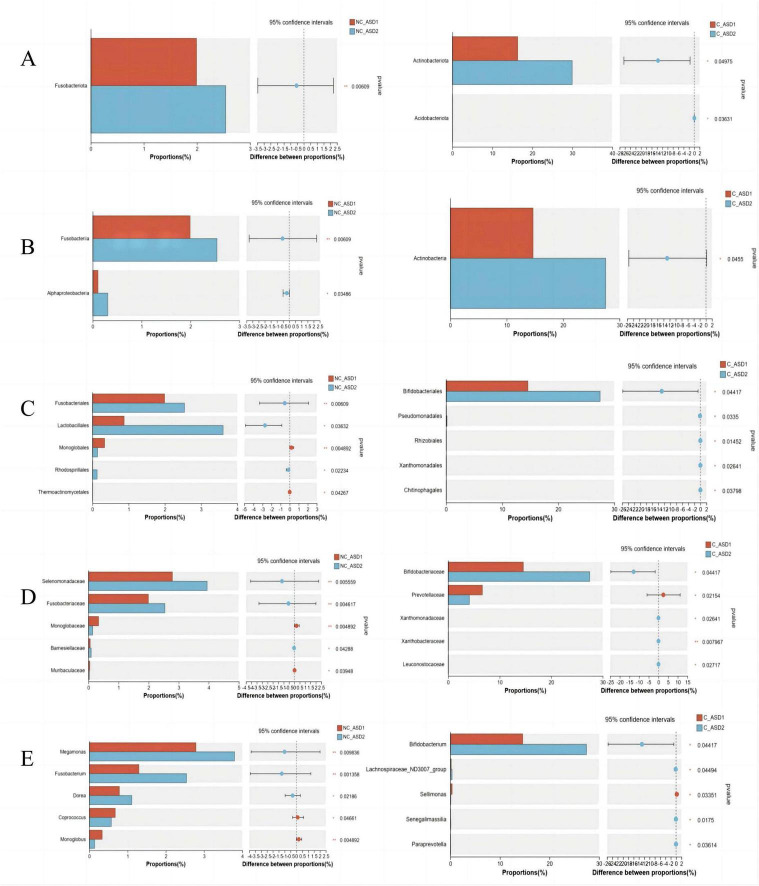
Analyze the differences in microbial composition between the non-constipation group and the constipation group, and identify the microorganisms that show significant differences between the two groups. **(A)** Phylum level. **(B)** Class level. **(C)** Order level. **(D)** Family level. **(E)** Genus level. The difference analysis between the two groups was primarily performed using the Wilcoxon rank-sum test. *P < 0.05; **P < 0.01; ns *P* > 0.05.

At the phylum level, following one WMT course, the relative abundance of *Fusobacteriota* significantly increased in the non-constipation group, but it did not account for a significant proportion of the overall community composition. In the constipation group, the relative abundances of *Actinobacteriota* and *Acidobacteriota* significantly increased, with *Actinobacteriota* rising from 16.34 to 29.97% (Figure 7A).

At the class level, following one WMT course, the relative abundances of *Fusobacteriia* and *Alphaproteobacteria* significantly increased in the non-constipation group, though both maintained low proportions in the overall community composition. In the constipation group, *Actinobacteria* showed a significant increase in relative abundance, with its proportion rising from 14.66 to 27.53% (Figure 7B).

At the order level, following one WMT course, the relative abundances of *Fusobacteriales*, *Lactobacillales*, and *Rhodospirillales* significantly increased in the non-constipation group, while *Monoglobales* and *Thermoactinomycetales* significantly decreased. In the constipation group, the relative abundances of *Bifidobacteriales*, *Pseudomonadales*, *Rhizobiales*, *Xanthomonadales*, and *Chitinophagales* significantly increased, with *Bifidobacteriales* rising from 14.59 to 27.46% (Figure 7C).

At the family level, following one WMT course, the non-constipation group showed significant increases in *Selenomonadaceae*, *Fusobacteriaceae*, and *Barnesiellaceae*, while *Monoglobaceae* and *Muribaculaceae* significantly decreased. In the constipation group, *Bifidobacteriaceae*, *Xanthomonadaceae*, *Xanthobacteraceae*, and *Leuconostocaceae* increased significantly, while *Prevotellaceae* significantly decreased. Notably, the abundance of *Bifidobacteriaceae* increased from 14.59 to 27.46% (Figure 7D).

At the genus level, following one WMT course, the non-constipation group exhibited significant increases in *Megamonas*, *Fusobacterium*, and *Dorea*, while *Coprococcus* and *Monoglobus* decreased significantly. In the constipation group, *Bifidobacterium*, *Lachnospiraceae ND3007 group*, *Senegalimassilia*, and *Paraprevotella* significantly increased, with *Sellimonas* significantly decreasing. The abundance of *Bifidobacterium* increased from 14.59 to 27.46% (Figure 7E).

LEfSe multi-level species difference analysis was performed on gut microbiota characteristics and differences from phylum to genus level before and after one course of WMT treatment for the two groups. Linear discriminant analysis (LDA) scores were used to show the influence of each species’ abundance on the differences, identifying communities or species responsible for significant differences. An LDA value > 3.5 indicates significant differences, with higher values reflecting greater impact.

**FIGURE 8:**
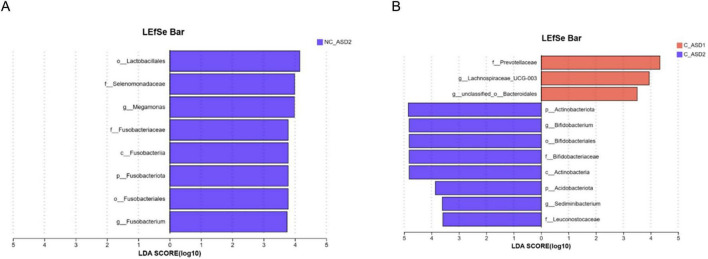
Changes and differences in gut microbiota between the two groups before and after WMT treatment. LefSe multi-level species difference analysis identified the most significantly different taxa in species abundance between the two groups: **(A)** non-constipation group, **(B)** constipation group. LefSe multi-level species difference analysis simultaneously employed the non-parametric Kruskal-Wallis sum-rank test, Wilcoxon rank-sum test, and Linear Discriminant Analysis for discriminative feature identification.

In comparisons of the gut microbiota before and after treatment between the non-constipation group and the constipation group, the top three highest LDA scores in the non-constipation group were from the NC_ASD2 group: *Lactobacillales* (4.15), *Selenomonadaceae* (3.99), and *Megamonas* (3.98), which are the differential species in the non-constipation group (Figure 8A). In the constipation group, the top three were from the C_ASD2 group: *Actinobacteriota* (4.85), *Bifidobacterium* (4.82), and *Bifidobacteriaceae* (4.82), which are the differential species in the constipation group (Figure 8B).

## 4 Discussion

Our previous study found that WMT treatment for ASD is effective, significantly reducing the scores of ABC, CARS, and SDSC, leading to obvious improvements in clinical symptoms of autism (Pan et al., 2022). This is consistent with findings from several current clinical studies. FMT has satisfactory long- term efficacy and safety for treating gastrointestinal and core symptoms in children with ASD (Li et al., 2021a; Li et al., 2021b; Ye et al., 2022).

Patients with ASD often suffer from gastrointestinal symptoms, with nearly half experiencing chronic gastrointestinal issues, including constipation, diarrhea, and bloating (Buie et al., 2010). However, whether the presence of constipation affects the efficacy of WMT treatment for ASD or influences the readjustment of gut microbiota in these children has not been studied. This research is the first to explore the impact of constipation on the clinical efficacy of WMT in children with ASD. Our study found that WMT significantly improved clinical outcomes for both ASD children with and without constipation before treatment. The ABC, CARS and SDSC scores in both groups decreased with the increase in WMT treatment sessions. However, did the efficacy of WMT treatment differ between the two groups? We compared the changes in ASD children with and without constipation before and after treatment and found no significant differences in the changes in CARS and SDSC scores between the two groups. There were significant differences in the ABC scores during the first and second treatment sessions, but after the third, fourth, and fifth sessions, there were no significant differences. This suggests that the efficacy was different in the early stages, but after multiple treatment sessions, the two groups showed no differences. Overall, in terms of mid-term efficacy, there is no difference in the effectiveness of WMT treatment for ASD between the non-constipation group and constipation group.

Research has shown that gastrointestinal symptoms are positively correlated with the severity of autism (Zhu et al., 2017). Chronic constipation, which persists over time, can increase intestinal inflammation, disrupt the intestinal mucosal barrier, and disturb the balance of gut microbiota (Huang et al., 2018; Shatri et al., 2023; Wang et al., 2022). This compromised internal physiological environment may aggravate ASD symptoms, underscoring the necessity to alleviate constipation while treating core ASD manifestations. Our findings indicate that after six courses of WMT treatment, the stools of patients with ASD in the constipation group approached normality. WMT as an emerging technique that functions by reshaping the gut microbiota, not only improves symptoms of autism but also alleviates constipation in children with ASD.

Constipation occurs in patients with ASD at a much higher rate than in normal children, and the onset is also earlier compared to other children (Kh and Gd, 2011; Lee et al., 2023; Restrepo et al., 2020). This high incidence is believed by some studies to be due to neurobehavioral factors rather than primary organic gastrointestinal causes (Ibrahim et al., 2009), while other research suggests it may be related to higher levels of gastrointestinal inflammation in children with ASD (Mathew et al., 2024). However, it is evident that gut microbiota play an important role in this. Constipation may alter the gut microenvironment by significantly reducing microbial richness and inducing abnormal levels of short-chain fatty acids, leading to lower diversity in the gut microbiota of those with constipation (Liu et al., 2019). A recent study independently explored the role of gut microbiota in patients with ASD and their comorbidities, finding nine bacterial taxa with significant relative abundance differences between non-constipated and constipated patients with ASD. Except for the Enterobacteriaceae family, all bacteria had significantly lower abundance in constipated patients with ASD (Fu et al., 2021). Additionally, six unique diet-related bacteria associated with constipation were found to have significantly reduced relative abundance in constipated patients with ASD, demonstrating a close link between constipation and the gut microbiota of patients with ASD.

In this study, by analyzing the gut microbiota diversity of both groups of patients with ASD, we found that those with comorbid constipation had lower gut microbial diversity at baseline compared to those without constipation. After one course of WMT treatment, the modulation of WMT on the gut microbiota of constipated patients with ASD was more pronounced, resulting in a more noticeable increase in diversity compared to non-constipated patients with ASD. In the intergroup difference analysis, we found that the relative abundances of Actinobacteriota, Actinobacteria, Bifidobacteriales, Bifidobacteriaceae, and Bifidobacterium significantly increased after one course of WMT treatment, with a considerable proportion of abundance. Meanwhile, in the LefSe multi-level species differential analysis, Actinobacteriota, Bifidobacterium, and Bifidobacteriaceae had the highest LDA scores in the constipation group, indicating that these three taxa are key differential species in the constipation group. Since Actinobacterioita, Bifidobacteriaceae, and Bifidobacterium represent a hierarchical relationship, the genus Bifidobacterium is the most critical among them. Previous research has suggested that Bifidobacterium possesses various beneficial functions, such as improving gut diseases caused by immune system disorders, inhibiting pathogen invasion in the gut and alleviating oxidative damage in the body (Ma et al., 2022; Turroni et al., 2021; Turroni et al., 2022). Bifidobacterium is also an essential beneficial bacterium for ASD. One study found that insufficient intake of Bifidobacterium is associated with an imbalance in gut microbiota structure, indicating that the colonization and transfer of Bifidobacterium may play a critical role in the development of ASD during early childhood (Coretti et al., 2018; Coretti et al., 2019). Animal experiments have shown that supplementation with Bifidobacterium can improve behavioral abnormalities and social deficits in autistic model rats, as well as regulate their gut microbiota (Abuaish et al., 2022; Abujamel et al., 2022). Two small-sample clinical studies have shown that supplementation with probiotic formulations, including Bifidobacterium, leads to significant improvements in both core ASD symptoms and gastrointestinal symptoms in patients with ASD (Sanctuary et al., 2019; Shaaban et al., 2018). Both animal experiments and clinical studies have demonstrated that an increase in the relative abundance of Bifidobacterium positively impacts the symptoms of children with ASD. Our research findings indicate that in the constipation group, the relative abundance of Bifidobacterium significantly increased after the first course of WMT treatment. Moreover, after the second course of WMT, the ABC scores in the constipation group showed a more significant decrease, suggesting a potential association between the two. However, since this study did not track changes in gut microbiota in the constipation group during subsequent courses two to five, further conclusions cannot be drawn.

Our study also found that in the constipation group, blood D-lactate levels significantly decreased after the first course of WMT compared to baseline, while there was no significant change in blood D-lactate levels in the non-constipation group. Statistical differences were observed when comparing the changes in blood D-lactate levels between the two groups after the first and second WMT courses, with the constipation group showing a greater reduction in D-lactate levels. D-lactate is a metabolic byproduct of bacterial fermentation, and its production is closely related to intestinal barrier function. When intestinal mucosal permeability increases, D-lactate produced by gut bacteria can enter the bloodstream through the damaged mucosa (Remund et al., 2023). The relationship between D-lactate and constipation may be reflected in changes in intestinal barrier function and gut microbiota balance (Chen et al., 2021). After the first course of WMT, the constipation group showed a significant increase in microbial diversity compared to the non-constipation group, with a notable rise in the relative abundance of Bifidobacterium. This increase may be attributed to the protective role of Bifidobacterium, which can form a bacterial barrier and promote the proliferation and differentiation of intestinal epithelial cells, thus actively contributing to the maintenance and enhancement of intestinal barrier integrity (Abdulqadir et al., 2023; Krumbeck et al., 2018). This suggests a close correlation between the significant increase in the relative abundance of Bifidobacterium and the notable reduction in D-lactate levels. Bifidobacterium can adhere to the surface of host intestinal epithelial cells, competitively inhibiting the invasion and colonization of intestinal pathogens, thereby improving the gut microbiota environment and significantly enhancing microbial diversity (Heiss et al., 2021; Xiao et al., 2021). Despite having a more imbalanced gut microbiota, the efficacy of WMT treatment in patients with ASD with constipation showed no significant difference compared to those without constipation, indicating that Bifidobacterium may play a crucial role in facilitating this process.

Our study also has some potential limitations. Firstly, as a retrospective study, we only measured the gut microbiota of ASD children before and after one course of WMT, leaving the effects of multiple courses of WMT on the gut microbiota in both the non-constipation and constipation groups unknown. Meanwhile, we only observed the short-term and medium-term changes in symptoms following WMT treatment, and further research is needed to explore the long-term treatment differences between the two groups. Furthermore, we cannot rule out the possibility of confounding factors in dietary habits or other behavioral therapies (Harris et al., 2021; Keller et al., 2021). Finally, due to the small size, our results should be validated through larger, prospective studies.

## 5 Conclusion

Our research indicates that after WMT treatment, both groups of ASD children showed significant improvement in core autism symptoms and sleep disturbances. With the increase in WMT courses, the stool shape in the constipation group returned to normal. In the early stages, there was a difference in treatment efficacy between the constipation and non-constipation groups; However, after multiple courses of WMT treatment, both groups exhibited no significant differences in efficacy. Although there was a notable increase in microbial diversity and a significant rise in the relative abundance of Bifidobacterium in patients with ASD with constipation following WMT, along with improvements in gut barrier function, the mid-term efficacy showed no differences between the non-constipation and constipation groups in WMT treatment for ASD. In summary, WMT appears to be an effective treatment for core and associated symptoms in children with ASD, including sleep disruption and gastrointestinal symptoms, regardless of the presence of constipation prior to receiving this intervention.

## Data Availability

The datasets presented in this study can be found in online repositories. The names of the repository/repositories and accession number(s) can be found at: https://www.ncbi.nlm.nih.gov/, PRJNA1199347.
